# COL4A1 Gene Mutation Masquerading as Cerebral Palsy: Report of a Rare Case

**DOI:** 10.7759/cureus.67351

**Published:** 2024-08-20

**Authors:** Shiji Chalipat, Jeevana Bollineni, Priyanka Shah, Vishwanath Kulkarni

**Affiliations:** 1 Pediatric Neurology, Dr. D Y Patil Medical College, Hospital and Research Centre, Dr. D Y Patil Vidyapeeth (Deemed to be University), Pune, IND; 2 Pediatrics, Dr. D Y Patil Medical College, Hospital and Research Centre, Dr. D Y Patil Vidyapeeth (Deemed to be University), Pune, IND

**Keywords:** cerebral palsy mimics, collagen type 4 alpha 1, epilepsy, mutation, vascular basement membranes

## Abstract

The Collagen Type 4 alpha 1 (*COL4A1*), is an important component of nearly all vascular basement membranes. Pathogenic mutation of this gene results in varied manifestations. In this report, we describe a two-and-a-half-year-old boy with an eventful perinatal period, global developmental delay, and epileptic spasms. Examination revealed microcephaly, nystagmus, and spasticity in limbs. Electroencephalogram showed multifocal epileptiform discharges and MRI brain demonstrated periventricular white matter changes, intracerebral bleeds, and porencephalic cysts. CT brain showed intracranial calcifications and screening for congenital infection was negative. The molecular genetic evaluation was later confirmed with a heterozygous mutation of the *COL4A1 *gene on exon 37 (variant - p.Gly1050Ala) with an autosomal dominant inheritance pattern. Currently, the child has developed drug-refractory epilepsy requiring polypharmacy and the ketogenic diet. *COL4A1* gene mutations are close mimickers of Cerebral Palsy, hence a high index of suspicion should be exercised while approaching a child with spastic quadriplegia in order to promptly diagnose and manage such children for a better neurological outcome.

## Introduction

The Collagen alpha-1(IV)(COL4A)-related disorder spectrum is an inherited genetic disorder, comprising two genes - *COL4A1* and *COL4A2*. Both of these genes are located on chromosome number 13 and are inherited in an autosomal dominant manner, with a small proportion (27%) of cases attributed to a de novo pathogenic variant [1]. These genes form the major structural component of type IV collagen, which in turn helps in cell migration, proliferation, cell differentiation, and survival. It was first described by Gould et al. in 2005 as a cause of perinatal cerebral hemorrhage in mice, however, in humans, it can present as a wide range of phenotypes [2]. 

Mutations in these genes manifest as weakening of basement vascular membranes thereby causing hemorrhage, small vessel diseases, familial porencephaly, and the HANAC (hereditary angiopathy with nephropathy, aneurysms, and muscle cramps) syndrome. A total of 67 families with *COL4A1* and *COL4A2* mutations have been described in the literature [1]. Clinically, small-vessel brain disease can present with a range of neurological symptoms. These may include infantile hemiparesis, seizures, single or recurrent hemorrhagic strokes, ischemic strokes, and isolated migraines with aura. Apart from this, two additional phenotypes include isolated retinal artery tortuosity and congenital cataracts. Here, we describe a two-and-a-half-year-old boy with global developmental delay (GDD) and epileptic encephalopathy secondary to a genetically proven *COL4A1* mutation.

## Case presentation

A two-and-a-half-year-old boy, with delayed attainment of milestones presented with epileptic spasms since one year of age. He was the firstborn out of a non-consanguineous marriage. He had an eventful perinatal period with a history of pregnancy-induced hypertension in the mother. He was born prematurely (at 35 weeks of gestation) with a birth weight of 1.7 kg, requiring admission to the neonatal intensive care unit in view of respiratory distress for 7 days.

All the milestones were attained late with a predominant motor developmental delay as opposed to cognitive delay. Since one year of age, the child had an onset of epileptic spasms. There was no significant family history. The child's anthropometric measures were appropriate for age. Examination revealed no obvious dysmorphic features and no neuro-cutaneous markers, however, the child had microcephaly (head circumference (HC) - 42 cm), nystagmus, and squint. Spasticity was present in all four limbs with exaggerated deep tendon reflexes. Other systemic examinations revealed no abnormality. The ophthalmologic evaluation revealed no cataracts or pigmentary retinopathy.

**Figure 1:**
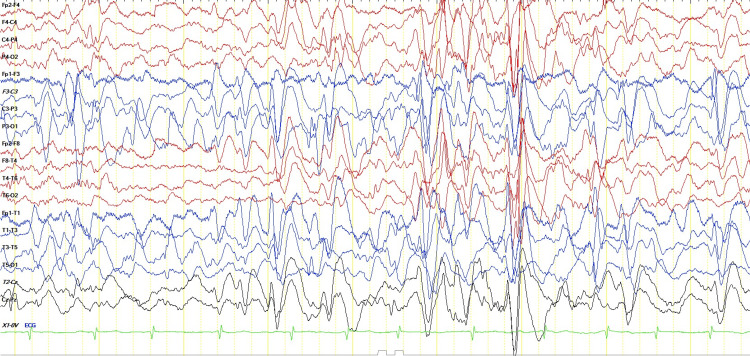
Electroencephalogram showing multifocal epileptiform discharges and generalized burst of spike and slow waves.

**Figure 2:**
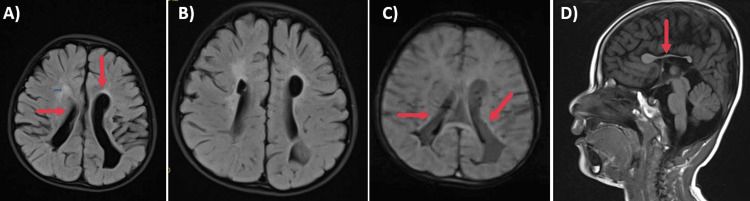
Magnetic resonance imaging (MRI) Brain A) MRI brain FLAIR axial section – showing asymmetric dilatation and squaring of occipital horns of lateral ventricles, hyperintensities along periventricular white matter and periventricular cysts. B) MRI brain FLAIR axial section – showing periventricular white matter hyperintensities and porencephalic cysts. C) MRI brain susceptibility-weighted image showing blooming with evidence of intraventricular bleed inside lateral ventricles. D) MRI brain - T1 sagittal section showing a dysplastic corpus callosum.

**Figure 3:**
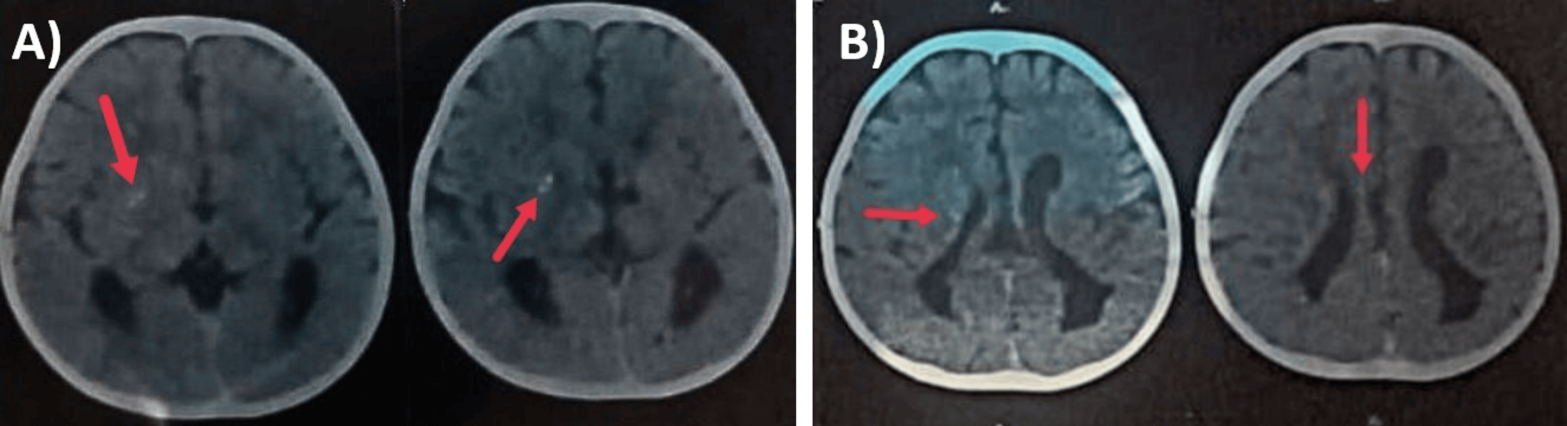
Computed Tomography (CT) of Brain A) CT Brain showing calcifications over basal ganglia; B) CT Brain showing calcifications in periventricular white matter

Electroencephalogram showed multifocal epileptiform discharges with intermittent bilaterally synchronous generalization and absence of sleep architecture (Figure 1). Magnetic resonance imaging (MRI) Brain showed periventricular hyperintensities, periventricular cysts, ex vacuo dilatation of lateral ventricles with squaring of occipital horns, white matter volume loss, and evidence of old intraventricular hemorrhages (Figure 2). So we went ahead with computed tomography (CT) brain to look for calcifications. It revealed multiple calcifications in bilateral periventricular white matter and basal ganglia (Figure 3).

Since the clinical presentation was like a static encephalopathy with perinatal events and MRI findings suggestive of sequelae of preterm brain injury, an initial differential diagnosis of spastic quadriplegia was considered. The presence of intracranial calcification hinted towards possibilities of congenital infections as well as genetic causes like Aicardi Gautier's syndrome and *COL4A1* mutations. Screening for congenital toxoplasmosis, rubella, cytomegalovirus (CMV), herpes simplex and human immunodeficiency viruses (TORCH) group of infections showed IgG CMV positivity which was nonspecific, and a negative urine CMV-PCR test. An ultrasonography of the abdomen was done to look for renal cysts, which revealed no abnormality and echocardiography also revealed a structurally normal heart. The child was started on anti-seizure medications with hormonal therapy for infantile spasms and neurorehabilitation was initiated. His spasms were drug-resistant, requiring polypharmacy (levitiracetam, valproate, clobazam, vigabatrin) and initiation of a ketogenic diet.

Subsequent whole genome sequencing revealed a heterozygous mutation in the *COL4A1* gene on exon 37 of chromosome 13 with an autosomal dominant inheritance. This was classified as a variant of uncertain significance. The clinical phenotype as well as the neuroimaging findings matched with the genotype even though it was a variant of unknown significance. MRI Brain and Sanger sequencing were recommended to the parents, after having been screened for renal cysts and cataracts. 

## Discussion

*COL4A1* mutations were initially described in humans as autosomal dominant porencephaly [2]. The phenotype not only involves the brain but affects the eyes and kidneys as well. The eye manifestations include cataracts, retinal artery tortuosities, and Axenfeld Rieger anomaly. The renal manifestations include renal cysts, hematuria, and renal failure [3]. About 67 families have been reported in the literature with a few cases reported from India as well [4]. However, many cases go underdiagnosed probably due to lack of testing, and are treated as non-genetic forms of static encephalopathy. Hence, more often than not *COL4A1 *mutations are known as ‘Cerebral Palsy’ mimics. The various neurological manifestations of this spectrum of disorder are usually secondary to intracerebral bleeds, porencephalic cysts, periventricular white matter involvement, and intraventricular hemorrhage [5,6].

Intracerebral bleeds can happen at any age starting from fetal life through childhood and adulthood. These can occur spontaneously or after minor trauma. Neurological abnormalities can vary from microcephaly, spastic quadriplegia, and infantile hemiplegia. The child was started on anti-seizure medications with hormonal therapy for infantile spasms and neurorehabilitation was initiated. Our case presented with microcephaly and spastic quadriplegia without cataracts. Seizures are very commonly reported in *COL4A1* spectrum disorders. Our patient presented with epileptic spasms which were drug refractory. Spasms have been reported in previous studies as well [5]. Epileptogenesis in patients with *COL4A* mutations can be explained by the development of cerebral vascular infarcts and microbleeds secondary to disrupted cerebral basement membranes, leading to increased neuronal excitotoxicity, changes in white matter, and impaired neuronal migration. This in turn leads to altered synapse formations, cortical malformations, gliotic changes, and white matter damage, thereby causing epilepsy, microcephaly, and developmental delays.

The neuroimaging findings of *COL4A1 *spectrum disorders usually include microinfarcts in the centrum semiovale and deep gray matter. Because the microvasculature in the basal ganglia region has sparse supporting tissue, there are higher incidences of microhemorrhages in this region [5]. Perinatal brain hemorrhages in fetuses and newborns have been described as the commonest manifestation of *COL4A1 *mutation [2]. This may be due to endothelial basement membrane weakness. Along with this, porencephalic cysts are often noted. These cysts are usually unilateral [7]. Our case had intraventricular hemorrhages as well as porencephalic cysts and periventricular white matter gliosis. Calcification over basal ganglia as well as periventricular white matter was also seen in our case. Intracranial calcifications especially in basal ganglia have been previously reported in cases with COL4A1 mutation [8,9]. The other neuroimaging findings described in the literature are schizencephaly, cortical developmental malformations, focal cortical dysplasia, hydranencephaly, and hydrocephaly [1,10].

The clinical and neuroimaging findings like periventricular leukoencephalopathy, intraventricular hemorrhage, and parenchymal bleeds very closely mimic preterm hypoxic-ischemic encephalopathy sequelae and intraventricular hemorrhage, and the calcifications mimic intrauterine infections in a child with static encephalopathy. In India, the prevalence of cerebral palsy is high with a high burden of prematurity, perinatal events, and congenital infections. Hence, we must have a high index of suspicion to diagnose this ‘Cerebral Palsy Mimic’.

## Conclusions

*COL4A1 *mutations are close mimickers of static encephalopathy with characteristic neuroimaging findings. The clinical clues, along with molecular markers of *COL4A1*, neuroimaging findings, and a high index of suspicion should prompt quick diagnosis and initiation of treatment for better neurological outcomes and quality of life in these patients as it will guide in genetic counseling.
